# Use of combined coagulation-adsorption process as pretreatment of landfill leachate

**DOI:** 10.1186/1735-2746-10-24

**Published:** 2013-03-21

**Authors:** Rajan Gandhimathi, Nalladurai Jegan Durai, Puthiya Veetil Nidheesh, Sreekrishnaperumal Thanga Ramesh, Subramaniam Kanmani

**Affiliations:** 1 Department of Civil Engineering, National Institute of Technology, Tiruchirappalli, Tamil Nadu, India

**Keywords:** Coagulation, Adsorption, Landfill, Leachate, COD

## Abstract

Landfill leachate is an important pollution factor resulting from municipal landfill sites. Physical and chemical processes are the better option for pretreatment or full treatment of landfill leachate. This article presents a combination of pre-treatment method (coagulation and adsorption) for leachate collected from municipal solid waste open dumping site. Physico chemical characteristics of stabilized and fresh leachate were examined. Coagulation process was examined by using alum and ferric chloride. A low cost adsorbent, fly ash was used for adsorption studies. Coagulation studies were carried out for fresh and stabilized leachate. Adsorption studies have been conducted for alum pre-treated stabilized leachate. Effect of coagulant dose, adsorbent dose, pH and contact time were carried out. The effective optimum coagulant dosages were 0.6 g/L and 0.7 g/L for alum and ferric chloride respectively for stabilized leachate and incase of fresh leachate 0.8 g/L and 0.6 g/L for alum and ferric chloride respectively. For the alum pretreated stabilized leachate, the maximum COD removal is 28% using fly ash adsorbent with equilibrium time of 210 min and optimum dose of 6 g/L. Overall COD removal efficiency of 82% was obtained by coagulation using alum and adsorption using fly ash for stabilized leachate. The results obtained showed that combined coagulation and adsorption process can be used effectively for stabilized leachate treatment.

## Introduction

Landfill leachate is an important pollution source originated in municipal landfill sites. Landfill leachate is defined as those aqueous streams generated as a consequence of rainwater percolation through wastes, biochemical processes in the wastes’ cells and the inherent water content of the wastes themselves [1]. Leachates may contain large amounts of organic matter, of which humic type constituents are an important group, as well as ammonia- nitrogen, heavy metals and chlorinated organic and inorganic salts [1]. The type of leachate depends on factors such as age and type of landfill, pH, and BOD_5_/COD ratio. Leachate may be classified as young (or raw), and stabilized (mature) [2]. Young leachates are generally low in pH (<6.5) and high in COD (>15,000 mg/L), biodegradable matters, BOD/COD ratio (0.5–1), and heavy metals (>2 mg/L); whereas old or stabilized leachates are usually high in pH (>7.5) and NH_4_–N (>400 mg/L) and low in COD (<3000 mg/L), BOD/COD ratio (<0.1) and heavy metals (<2 mg/L) [3]. The discharge of landfill leachate can lead to serious environmental problems as they may percolate through soils and sub soils, causing extensive pollution of ground and surface waters if they are not properly treated and safely disposed [4]. Leachate becomes ahead of wastewaters as being the most difficult to treat as it is a wastewater with a complex and widely variable content generated within a landfill. Therefore, many pretreatment and combined treatment methods have been proven to treat leachate [5].

Conventional landfill leachate treatments can be classified into three major groups: (a) leachate transfer to a municipal wastewater treatment plant where it is subject to combined treatment with domestic sewage, (b) biodegradation (aerobic and anaerobic) processes and (c) chemical and physical methods such as chemical oxidation, adsorption, chemical precipitation, coagulation/flocculation, sedimentation/flotation and air stripping [6]. Biological treatment is primarily used to stabilize degradable organic matter in the young and middle aged leachates [7]. However, they generally fail to treat a leachate with a rather low BOD_5_/COD, or high concentration of toxic metals [8]. Hence, physico-chemical processes are mostly used for pretreatment or full treatment for this type of landfill leachate [9]. Physico-chemical processes are used for the pretreatment of young leachate to make it amenable to biological treatment and to hydrolyze some refractory organic compounds found in leachate from older landfills [7].

Various physical-chemical processes such as adsorption [1,10], coagulation/flocculation [2-4,11], Fenton process [12], electro chemical oxidation [8], electrocoagulation [5] etc. were applied successfully for the treatment for landfill leachate. Combination of the above process reduces the drawbacks of each single process. The removal efficiency of COD from leachate by adsorption process is very low [10]. The leachate treatment by adsorption is effective at low COD values. At the same time, chemical coagulation works well for leachate pre-treatment. By combining these two processes, an effective COD removal from landfill leachate can be achieved [13]. Thus combination of these two processes may be a promising technology for the pretreatment of leachate.

The aim of this study was to study the feasibility of the combined coagulation and adsorption process as a pre-treatment for COD removal in landfill leachate. Leachate was collected from open dumping site at Ariyamangalam, Tiruchirappalli city, Tamil Nadu, India. The Ariyamangalam dumping site in Tiruchirappalli Corporation has been in operation since 1967, covering a total surface area of 47.5 acres. The dumping site is located at an altitude of 75.88 m above Mean Sea Level (MSL) with latitude of 10°48’ N and longitude of 78°43’ E. In this dumping site solid wastes (approximately 400 tonnes/day) are deposited with a depth of 3 to 5 m. In this dump yard, waste is disposed without segregation and compaction. No daily or intermediate cover is placed over the deposited waste. Access to the site is freely available to all and the site is regularly utilized by rag pickers, scavenging for recyclable materials, and by a variety of animals, including water buffalo, cattle, pigs, and dogs, scavenging for food waste. There will be no lining at the bottom of the dump yard. The leachate produced due to the biological decomposition of municipal solid waste and entry of rainwater percolates through the soil and eventually pollutes the groundwater in the surrounding areas.

Alum and FeCl_3_ were used as potential coagulants for the present study. Effect of coagulant dose on COD removal was examined and compared. The specific aim of this work was to study the combined effect of above two processes for COD removal from stabilized leachate. Fly ash “C” was used as adsorbent for this study. Fly ash is a waste material produced abundantly as a byproduct from industries such as thermal power plants, steel mills etc. Fly ash was reported as a good adsorbent for treating leachate [10,14]. Effect of fly ash dosage and contact time on COD removal from leachate also investigated.

## Materials and methods

### Leachate

Leachate samples collected from the open dumping site were filled in plastic container, transported to the laboratory and stored at 4°C and analyzed within 2 days. A total of 20 leachate samples were collected for monitoring purpose. Out of which 11 stabilized leachate samples were collected in old dumping area (age > 3 years), whereas the remaining 9 fresh leachate samples were collected near to the new dumping area (< 2 years). The parameters such as pH, COD, BOD, total hardness, electrical conductivity, total dissolved solids, major cations such as Ca^2+^, Mg^2+^, and iron, major anion such as Cl^-^ were determined. All the analyses in this study were repeated two or three times until concomitant values were obtained, and all the tests were carried out according to the standard methods [15].

### Pretreatment of leachate by coagulation process

Coagulation studies were performed in a conventional jar-test apparatus, equipped with six beakers of 1 litre volume at room temperature. The experimental process consist of three subsequent stages: the initial rapid mixing stage took place for 3 min at 160 rpm, followed by a slow mixing stage for 17 min at 40 rpm. Stirring was then discontinued and the sludge was left to settle for an hour. After the settling period, the supernatant was withdrawn from the beaker and was analyzed and wet sludge volume was estimated from the sludge level deposited at the bottom of the glass beakers after 24 h. Coagulants such as alum (Aluminium sulphate) and Ferric Chloride were supplied in liquid state. Mother solution containing 10 g Fe^3+^/L, 10g Al^3+^/L were prepared for coagulation experiment. The experiments were carried out without prior pH adjustment using different coagulant dosages (0.2 to 1 g/L). Supernatant solution after coagulation process for different coagulant dosage was analyzed for their residual COD concentration and change in pH.

### Removal of COD in the pre treated stabilized leachate by adsorption

Adsorption batch study has been carried out using fly ash ‘C’ for alum pretreated stabilized leachate. The adsorbent was obtained from Neyveli Lignite Corporation Limited, Neyveli, Tamil Nadu. Characteristics of the fly ash were reported in our previous work [10]. Kinetic study was conducted with the known dose of adsorbent (0.025 g) for the 50 mL of pretreated leachate samples. The samples were shaken at an agitation rate of 150 rpm. The samples were taken out at different intervals in between 5 and 300 minutes. The sorbent solution mixtures were then centrifuged at 6000 rpm for 5 minutes and the supernatant was analyzed for the residual COD.

The batch isotherm studies were carried out by shaking a series of bottles containing various amounts of fly ash dosage (0.05 to 0.5 g) in 50 ml of pretreated leachate samples. Fly ash particles were dried at 110°C for 2 hours before the batch experiment. The samples were stirred at room temperature at 150 rpm for equilibrium time, then their content was centrifuged at 6000 rpm for 5 minutes and the supernatant liquid was analyzed for changes in the solution pH and residual COD.

## Results

**Figure 1:**
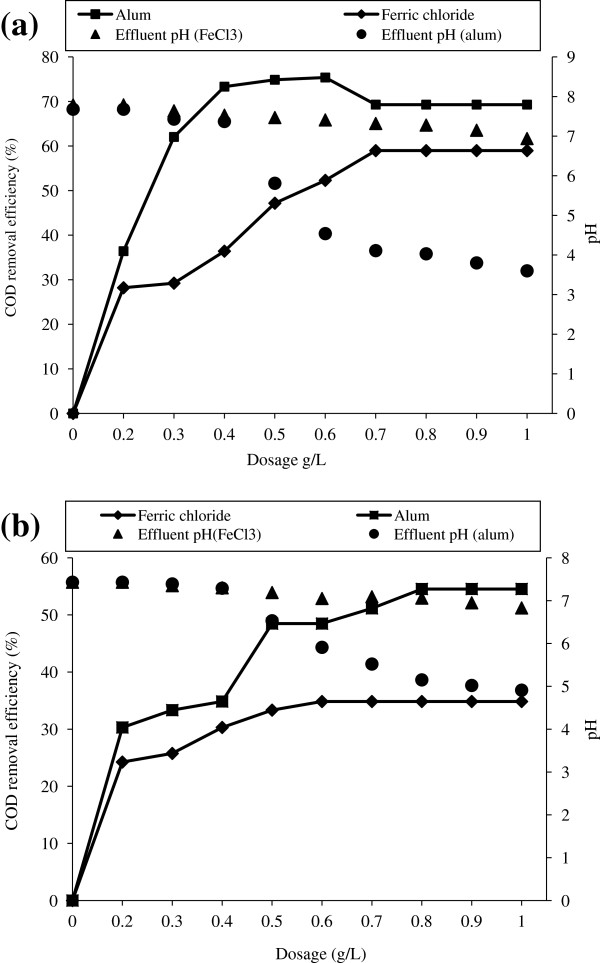
COD Removal and pH variation for different dose of ferric chloride and alum for (a) stabilized leachate (b) fresh leachate.

**Figure 2:**
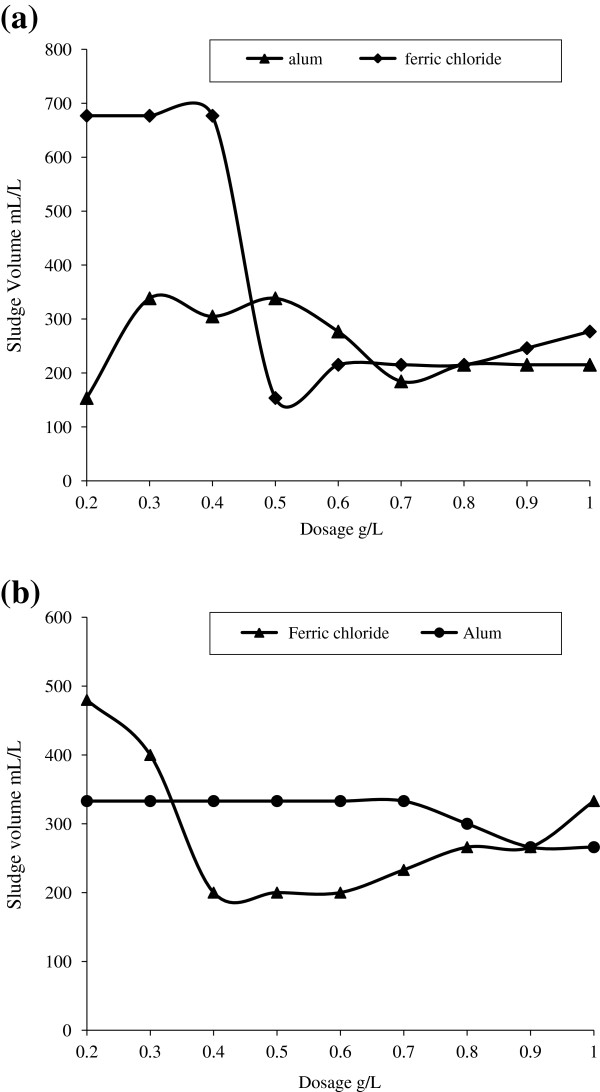
Amount of sludge produced for different dose of ferric chloride and alum for (a) stabilized leachate (b) fresh leachate.

**Figure 3:**
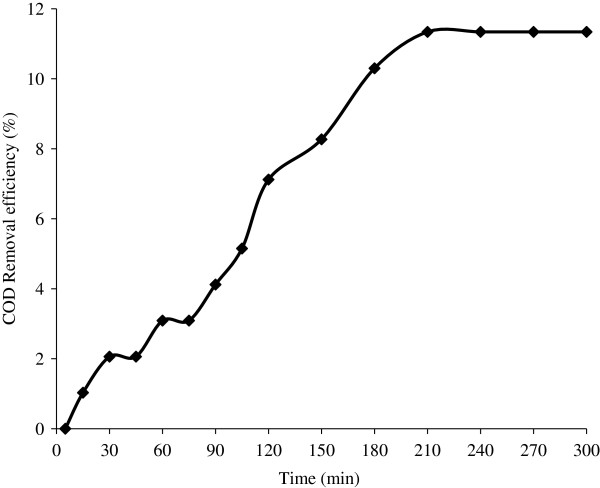
Effect of contact time on COD removal.

**Figure 4:**
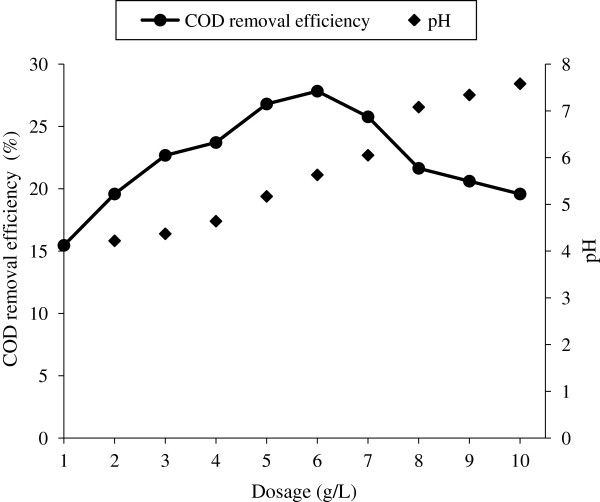
COD Removal and variation in pH for different dose of fly ash.

The characteristics of fresh leachate and stabilized leachate are given in Table 1. And the leachate characteristics used for pre treatment with different coagulants are reported in Table 2. Removal efficiency of COD for different dosage of ferric chloride and alum without prior adjustment of pH for stabilized and fresh leachate is shown in Figure 1. The variation in pH due to addition of coagulants is also represented in Figure 1. Amount of sludge produced for different dose of FeCl_3_ and alum is shown in Figure 2. The optimum conditions for both alum and FeCl_3_ coagulation is given in Table 3. COD removal for constant fly ash dose of 0.5 g/L at different time period is shown in Figure 3. COD removal and effluent pH for different dose of fly ash for equilibrium time is shown in Figure 4.

**Table 1:** Typical range of leachate characteristics

**S. No.**	**Parameter**	**Fresh leachate**			**Stabilized leachate**		
		**Value range**	**Average**	**Standard deviation**	**Value range**	**Average**	**Standard deviation**
1	pH	6.96-7.77	7.44	0.268	7.68-8.33	8.03	0.195
2	Electrical conductivity (μs)	11130-65000	31126.67	18579.06	6810-40300	21980	12217.05
3	Total dissolved solids (mg/L)	10000-47000	23888.89	12404.08	6000-29000	17181.9	8109.47
4	Chlorides (mg/L)	1000-6500	3276.99	2385.80	1000-6500	3450.76	2049.82
5	Total alkalinity (mg/L as CaCO_3_)	4500-19000	10944.44	4914.54	2500-11000	6409.09	3096.91
6	Total hardness (mg/L as CaCO_3_)	4000-11000	8500	2061.55	4500-8500	4772.72	1472.47
7	Calcium (mg/L)	1200-3000	1911.11	600.92	400-1800	1000	464.75
8	Magnesium (mg/L)	250-1500	904.5	434.31	250-1100	552.27	299.41
9	BOD_5_ (mg/L)	3300-43328	19884.27	14947.05	60-3000	936.14	1006.20
10	COD (mg/L)	6240-66240	30791.11	22383.15	1024-19200	8951.27	6011.64
11	Iron (mg/L)	14-600	261.88	198.15	40-82	126.66	134.86

**Table 2:** Characteristics of leachate before pretreatment

**S. No.**	**Parameters**	**Fresh leachate**	**Stabilized leachate**
1	pH	7.4	7.87
2	Total dissolved solids (mg/L)	20000	13000
3	Electrical conductivity (μs)	25800	14700
4	Chlorides (mg/L)	2500	3498
5	Total alkalinity (mg/L as CaCO_3_)	10,500	6000
6	Total hardness (mg/L as CaCO_3_)	5000	4000
7	Calcium (mg/L)	800	600
8	Magnesium (mg/L)	729	607
9	BOD_5_ (mg/L)	10812	1952
10	COD (mg/L)	16896	6240

**Table 3:** Optimum coagulation results

**Coagulant**	**Stabilized Leachate**				**Fresh Leachate**			
	**Optimum dose (g/L)**	**COD Removal efficiency (%)**	**Effluent COD at optimum dose (mg/L)**	**Effluent pH at optimum dose**	**Optimum dose (g/L)**	**COD removal efficiency (%)**	**Effluent COD at optimum dose (mg/L)**	**Effluent pH at optimum dose**
Alum	0.6	75	1560	4.11	0.8	55	7603.2	5.15
FeCl_3_	0.7	59	1872	7.28	0.6	35	10982	7.05

## Discussions

### Characteristics of leachate

To study the leachate characteristics, leachate samples from the actual leachate streams in the solid waste dumping site were collected and analyzed. From the Table 1, it can be observed that the fresh leachate sample possesses very high concentration of chemical parameters except pH, when compared to stabilized leachate samples. The range of COD varies from 6240 mg/L to 66240 mg/L for fresh leachate and 1024 mg/L to 19200 mg/L for stabilized leachate samples. The BOD_5_/COD value of fresh leachate is 0.52-0.65. This is in the range reported by Ghafari et al. [3] for fresh leachate. The BOD_5_/COD ratio of stabilized leachate varies from 0.06 to 0.15. The composition of fresh leachate can be considered as characteristic of acidic decomposition. Similarly, the high value of conductivity of 11130 – 65000 μ Mho/cm indicates the presence of large amount of dissolved inorganic materials in the fresh leachate samples. The pH value of the fresh samples was observed in the range of 6.96 – 7.77, but the older samples were amber colored and alkaline with pH range of 7.68 to 8.33. This may be attributed to the decrease in the concentration of free volatile acids due to anaerobic composition, as fatty acids can be partially ionized and contribute to higher pH values. Alkaline pH is normally encountered at landfills, 10 years after disposal [16]. In addition to high organic compound concentration, both leachates contain high concentrations of inorganic substances such as chlorides, calcium, magnesium and iron. The presence of magnesium in the leachate is due to the disposal of construction waste along with MSW [17]. Fresh leachate samples were found to contain relatively high concentrations of iron (14 to 600 mg/L), whereas the old samples were found to have values less than 82 mg/L. This reduction represents the dilution effect with respect to age of the dumping site.

### Pretreatment of leachate using coagulation

The effect of coagulant dose was studied by varying the coagulant dose from 0.2 to 1 g/L and the removal efficiency of COD, pH of the effluent and sludge volume was analyzed. From Figure 1(a) it is observed that the COD removal efficiency increases with the increase of dose from 0.2 to 0.6 g/L for alum and 0.2 to 0.7 g/L for ferric chloride and thereafter remains constant for stabilized leachate. From Figure 1 (b) it can be observed that the fresh leachate COD removal efficiency increases with the increase of dose from 0.2 to 0.8 g/L for alum and 0.2 to 0.6 g/L for ferric chloride and remains unchanged thereafter. The optimum dose of ferric chloride was 0.7 and 0.6 g/L with the COD removal efficiency of 59 and 35% for stabilized and fresh leachate respectively. For alum, the removal efficiency becomes maximum of 75 and 55% at 0.6 and 0.8 g/L for stabilized and fresh leachate respectively. From the Figure 1 (a) and 1 (b), it was found that alum has better COD removal efficiency than that of FeCl_3_. This is due to difference in hydrolysis of these coagulants. The mechanism of both alum and FeCl_3_ coagulation was controlled by pH, coagulant dosage and colloid concentrations [18]. For Aluminium - H_2_O system, the equilibrium concentration was controlled by Al (OH)_3_ and Al(OH)_4_^-^ concentration at alkaline medium (pH >7.2) and Al (OH)_3_ and Al_13_(OH)_34_^5+^ concentration in the pH range 7.2 to 5.6 [19]. Similarly for Iron- H_2_O system the equilibrium concentration was controlled by Fe (OH)_3_ and Fe(OH)_4_^-^ concentration at pH > 10, concentration of Fe(OH)_3_ for pH in between 7 and 10, concentration of Fe (OH)_3_ and Fe(OH)^2+^ for pH in between 3.5 and 7 and concentration of Fe^3+^ at pH < 3.5. For both the coagulants the process was occurs due to (i) adsorption and charge neutralization and (ii) enmeshment in sweep flocculation [18].

From the Figure 1 (a), alum dose up to 0.3 g/L, the COD removal is due to enmeshment in Al (OH)_3_ precipitate. In that region the change in pH is insignificant (7.8 to 7.37). But after that there is a significant change in pH (7.37 to 4.11) up to an alum dosage of 0.6 g/L. The COD removal at this point may due to both charge neutralization (Al_13_(OH)_34_^5+^) and enmeshment of pollutants on Al(OH)_3_. By increasing the dose beyond the 0.6 g/L, the removal efficiency decreases due to reduction in pH (4.11 to 3.6). For fresh leachate (Figure 1 (b)), COD removal mechanism is similar to the stabilized leachate. It can be observed that the change in pH is insignificant after the optimum dose. This may be due to high amount of alkalinity in the fresh leachate when compared to stabilized leachate (Table 2). For FeCl_3_ coagulation (Figure 1), there was an insignificant decrease in pH throughout the process. The pH was maintained near neutral condition. So the COD removal by FeCl_3_ is due to enmeshment of pollutants on Fe(OH)_3_. But the formation rate of Fe(OH)_3_ is very less compared to Al(OH)_4_^-^ and Al_13_(OH)_34_^5+^[18]. Due to that the less COD removal efficiency has been observed in FeCl_3_ coagulation.

Amount of sludge produced for different dose of ferric chloride and alum for stabilized and fresh leachate after 24 hr was presented in Figure 2. From the Figure 2 (a) and 2 (b), it was found that the increase in dose decreases the sludge production for ferric chloride compared to alum. In case of alum, the maximum sludge production was found to be 338.4 mL/L for the dose of 0.5 g/L and 333 mL/L for the dose of 0.7 g/L for stabilized leachate and fresh leachate respectively.

### Removal of COD in the pre treated stabilized leachate by adsorption

Removal of COD in the pretreated stabilized leachate by alum coagulation (COD = 1560 mg/L; pH = 4.11) has been carried out by adsorption process using fly ash ‘C’. From Figure 3 it was found COD removal is of 3%, 7.2% was obtained at 60 min, 120 min respectively and 11.34% COD has been removed at 210 min and remains constant. The time required to attain this state of equilibrium is termed the equilibrium time, and the amount of pollutant adsorbed at the equilibrium time reflects the maximum adsorption capacity of the adsorbent under those operating conditions [20]. Thus the equilibrium time was taken as 210 min.

From the Figure 4, it has been observed that the COD removal is15% for 1 g/L and increases to 27% for 6 g/L of fly ash, which is due to the increase in adsorbent surface area of the adsorbent [21]. By increasing the fly ash dose beyond the 6 g/L, the COD removal efficiency decreases. The pH of the leachate also increases (4.11 to 7.6) with increase in fly ash dose. The point of zero charge of fly ash used in the present study is 6.9 [10]. At pH less than 6.9, the surface of fly ash is predominated by positive charges. While at pH greater than 6.9 the surface is predominated by negative charges. The COD removal efficiency is maximum below the point of zero charge and decreases after the point of zero charge. This is due to repulsion between colloidal organic matter and negatively charged fly ash surface. The active sites present on the adsorbent also decreases due to heavy metal adsorption (cations) over the point of zero charge.

The COD removal efficiency of 75% (6240 to 1560 mg/L) was obtained by coagulation using alum and removal efficiency of 27% (1560 to 1143 mg/L) by adsorption using fly ash. Thereby, the overall COD removal efficiency of 82% has been obtained for stabilized leachate by combined coagulation and adsorption process. This indicates that this combination can be used as pretreatment or full treatment of stabilized landfill leachate.

## Conclusions

Based on the results presented above, the following conclusions can be drawn for the combined treatment of leachate.

• From the Coagulation experiment it was found that the removal efficiency of COD using alum is 75% while for ferric chloride it was 59% without prior adjustment of pH for stabilized leachate and it was found that the removal efficiency of COD using alum is 55% while for ferric chloride it was 35% without prior adjustment of pH for fresh leachate.

• The effective optimum coagulant dosages were 0.6 g/L and 0.7 g/L for Alum and ferric chloride respectively for stabilized leachate and incase of fresh leachate 0.8 g/L and 0.6 g/L for Alum and ferric chloride respectively.

• The COD removal efficiency was achieved 53% for ferric chloride at pH of 2 and goes on decrease in the removal efficiency from acidic to alkaline pH, for alum the removal is 55% at pH 8.

• The sludge production is more for ferric chloride and less for aluminium sulphate.

• Optimum time of 210 min, optimum dose of 6 g/L the maximum COD removal is 28% using fly ash for alum pre treated stabilized leachate.

• Overall COD removal efficiency of 82% was obtained by coagulation using alum and adsorption using fly ash for stabilized leachate.

## Competing interests

The authors declare that they have no competing interests.

## Authors’ contributions

NJD and SK carried out the experiments under the guidance of RG and STR. PVN, RG and STR compiled the experimental data in journal format. All authors read and approved the final manuscript.
